# Birmingham Hospital Saturday Fund

**Published:** 1894-06-16

**Authors:** 


### The Institutional Workshop.

### HOSPITAL ADMINISTRATION.

### BIRMINGHAM HOSPITAL SATURDAY FUND.

Mr. Smedley is in a fighting humour, and, not content with attacking Mr. Burdett for venturing to suggest that the working men of Birmingham get back from the medical charities more than they give, he pours out upon The Hospital an avalanche of words to prove that we are thoroughly unfair in our treatment of the subject. Our sin in the matter appears to be that we supported Mr. Burdett in the testimony which he gave to the services rendered to the Birmingham Hospital Saturday Fund by the late Mr. Sampson Gamgee, Mr. G. F. Muntz, and Mr. Geoige Dawson. This really is outside the question which is of chief interest to the present generation, viz., as to the bearing of working-class contributions on hospital administration; but when Mr. Smedley chose to round upon the founders of the Fund, stating, as he did in The Hospital of May 19th, that "Mr. Sampson Gamgee was undoubtedly the founder of the movement in Birmingham, but whatever success it has attained to-day has not been built on the foundations he laid, but on foundations laid in direct opposition to his advice," we felt bound to remind our readers that the vital principles in the scheme were there from the beginning, that to its founders the credit of its success was largely due, and that among the men interested in its inception were those whose names we gave.

Mr. Smedley is greatly concerned about the names we mentioned in conjunction with that of Sampson Gamgee, and gives a list of persons in his view more worthy of honourable mention. Perhaps he will forgive us for suggesting that even his list is but an imperfect record of the services of innumerable men who, whether as presidents, or local secretaries, or workers in the factories collecting the humble penny, gave their services freely to help forward the success of this great Hospital Saturday movement. It was obvious, however, that Mr. Burdett, in referring to the founders, merely mentioned the names of a couple of men who were known to have been interested in the very beginning of the movement, and in no way suggested that others were not similarly engaged; and for all the attempt made by Mr. Smedley to minimize their influence, it remains the fact that the very address by Sampson Gamgee, to which Mr. Smedley refers, opens by the statement that the first Hospital Saturday collection "had been projected at a working men's meeting presided over by George Dawson," and also so interested was Mr. Muntz in the scheme, not merely in the hospitals but in the efforts of the working men, that as soon as the accounts of the first collection were made up, he sent a cheque for £500, in order that the whole of the contributions should go intact to the objects for which they had been collected. There can be no doubt, then, that Mr. Burdett was correct in mentioning these two names in association with that of Mr. Gamgee, just as there equally can be no doubt in the mind of any man of common sense that he had no intention of making invidious comparisons or of withholding credit from those to whom it was due.

These, however, are minor points; what we are concerned about is the attack made upon the memory of the late Mr. Sampson Gamgee by the assertion, reiterated in various forms, that the success of the Fund has not been built on the foundations he laid, but on foundations laid in direct opposition to his advice. No doubt after Mr. Gamgee ceased to be secretary to the scheme it fell back somewhat from its former promise, and when in 1878 new blood was put into the concern, and fresh methods of collection were introduced, we can quite understand the belief of those who saw prosperity attending the penny-a-week system that this plan was the key to progress—that this system was the real foundation on which success was to be built. But the foundation really lay deeper. Method of collection was a detail; and although Gamgee much objected to the penny-a-week as being less obviously a labour sacrifice, and thus less [illegible]obling to the giver than the devotion of a d[illegible]s work, he by no means made the method of collection an essential. He himself said, "As one means of promoting the Hospital Saturday collection the penny-a-week system has been, and is, very useful. Nothing should be done to injure it—all that is possible to supplement it." The system as a means has been a mine of wealth, but the penny-a-week contribution is a plan far older than the Hospital Saturday, and one that has been employed by many an enterprise which has ended in utter failure. It is by no means the foundation of success.

The aims of the movement from the beginning were that the gifts should be voluntary, that they should be free, given by the strong and healthy for the benefit of the helpless and the sick; that the working classes should co-operate in their distribution and in the checking of hospital abuses; that "the influence of the working classes should be felt in hospital administration in direct proportion to the growth of hospital support"; that they should allot money for certain purposes approved by the Hospital Saturday Committee; that conferences of committees of different towns should be held. These are points picked out from a cursory inspection of Mr. Gamgee's lecture on the "Origin and Future of Hospital Saturday," and surely they represent the Birmingham Hospital Saturday as it now is.

To say that success has been built on foundations laid in direct opposition to Gamgee's advice, merely because a penny a week was found to accord better with the habits of a weekly wage population than did a larger sacrifice once a year, is to magnify details and forget the principles which touched the sympathy, the goodwill, and maybe the ambition of the working classes of Birmingham, namely, the voluntary offering, the free gift, the participation by the working classes in its distribution, the sense of doing the whole thing themselves and of getting the whole credit of it.

The great offence which has roused Mr. Smedley to so many words, has to do with modern times rather than with history, and lies in the mere suggestion that the working people, as a class, not as individuals, get a good return for their Hospital Saturday contributions. Now, according to the official report, the free gift of the Hospital Saturday Fund to the hospitals was £10,000, and £1,000 to their own convalescent home, and according to Mr. Smedley's statement, the expenditure of the fifteen medical charities which participate in the collection was £55,000. The actual expenditure on the relief and maintenance of patients, and on administration, on which the award was made was £45,971, and we may take for granted that the great mass of this money was spent on people who may fairly be ranked among the working classes. It would appear then that Mr. Burdett's assertion is correct; that even so far as the £10,000 is a free gift it is, as a matter of class, a gift to themselves, and that the added money is very great.

If people give £11,000 and receive nearly £46,000, besides the use of an enormous capital sunk in buildings, they get a very good *quid pro quo* ; and, greatly as we admire the good feeling which has led the Birmingham artisans to make this splendid contribution to the medical charities of their town, the time is clearly yet far off when these institutions can be looked on as co-operative affairs, or when the working man who receives their charity can feel that as a member of a class he pays for what he gets.

Mr. Smedley protests that this is unfair, and says that the contribution is £25,000 instead of £11,000. We are very glad to hear it, but we fail to find any such record in the report of the Hospital Saturday Fund. Of course he means that besides their free gift the workpeople subscribe another £14,000 direct to the hospitals in return for tickets. But that is exactly the point on which we state that Mr. Burdett asked for information. He further urged that until the accounts of the Hospital Saturday Fund included the whole of the contributions made by the working classes to the hospitals from every source, it was open to question how far, if at all, the £11,000 could be regarded as a free gift, although no tickets were actually issued in exchange for this money.

It is worth while here to draw attention to a fact which does not appear in the report of the fund, but is made clear by the statements of Mr. Smedley, that the penny-a-week subscription, of which so much is made as a foundation of success, is not paid to the Hospital Saturday Fund at all, but to a shop fund out of which direct subscriptions are first given to various hospitals in return for tickets, and from which registration fees are paid to those hospitals which demand them. All these payments are for the use of the working men, and only out of the balance left is the free gift voted to the Hospital Saturday Fund.

The whole question then whether this is really a free gift, a charity, not for the use of the givers, but of others less fortunately placed, depends on how far the direct subscriptions, or in other words, the prices paid for the tickets, are calculated on a proper basis.

If on the average the money paid for the ticket covers the cost of the patient, this £10,000 given by the Birmingham artisans is a pure charity, available for the good of others exactly as are the contributions given by ordinary subscribers, who also expect no return; and if that is the case, we offer our homage to the working men of Birmingham, and join willingly in the pæans raised in honour of the Saturday Fund. If, however, every sum voted to the fund is but the residue of a still larger sum expended in the purchase of tickets at such a price as to involve the hospitals in a loss, the matter assumes a different complexion.

We have already said that on an average the in-patient ticket at the General or the Queen's Hospital involves a loss of 18s., and that the registration fee of 1s. entails a loss of 6d.

Now if, as Mr. Smedley maintains, the contribution of the working classes amounts to £25,000, the balance of this, after deducting the free gift, can only be spent in direct subscriptions or purchase of tickets at this ruinous rate. It is clear, therefore, that the larger the amount subscribed by the working classes under such conditions the greater the drain upon the hospital funds, and the less the proportion of the free gift available for the proper purposes of the charity. We have gone into the matter carefully, and we have no doubt that the working people of Birmingham do give a free gift to the medical charities, but certainly not one amounting to £10,000. Under these circumstances where can be the objection to the introduction of a system of book-keeping, for such the Potteries system really is, by which the hospitals could discover which workshops are a drag on their resources, and which a help. Without some such system it is impossible for the managers of the fund to know whether any given sum received represents a donation or a call. The offerings of two workshops may be the same, but while one may be a free gift the other may be accompanied by such a purchase of tickets as to represent a large expenditure, and we can see no excuse for the organisers of so large a trust keeping themselves in ignorance of such important details.

In regard to Mr. Smedley's very positive assertion that at Wolverhampton the Hospital Saturday collections are managed by the hospital officials, we have made further inquiry, and are able to state that the collections are made under the sole control of a special committee elected by the different workshops in the town and district. The weekly board of the hospital are *ex officio* members of the committee, but they always leave the management entirely to the workpeople's representatives; Mr. Smedley would appear, therefore, to be under an entire misapprehension in this matter. But what are we to say of his figures regarding the population from which the subscriptions are drawn? Wolverhampton is a town of 82,620 inhabitants; he finds, however, that an odd sum of £22 came to the fund from Walsall, so he adds to the contributing population all the 71,791 inhabitants of that town, and in the same way all the 45,740 inhabitants of Dudley on the strength of a stray subscription of two guineas. Such is the mistaken application of statistics.

Mr. Smedley again says that the Wolverhampton Hospital Saturday is a mere provident institution; so much is paid by each manufactory and so many tickets are received in exchange. He should, perhaps, have added that the tickets so received give no right to indoor treatment, and that it is quite possible that a ticket subscription on these lines may be less exhausting to the charity than a combined ticket subscription and free gift on the system pursued at Birmingham.

Anyone who will read our previous articles on this subject will surely admit that we have spoken in terms of the highest admiration of the good feeling which has prompted the splendid contribution of the working men of Birmingham to the medical charities of the town, and of the untiring energy by which the organisation has been maintained. We should, however, be neglecting our duty as exponents of progress in hospital administration did we not emphasise the importance of the question asked by Mr. Burdett—How far can the Birmingham Saturday Fund be properly called a free gift?

